# A non-destructive enzymatic method to extract DNA from arthropod specimens: Implications for morphological and molecular studies

**DOI:** 10.1371/journal.pone.0192200

**Published:** 2018-02-01

**Authors:** Daubian Santos, Guilherme Cunha Ribeiro, Aline Diniz Cabral, Márcia Aparecida Sperança

**Affiliations:** 1 Universidade Federal do ABC,Centro de Ciências Naturais e Humanas, Santo André, São Paulo, Brazil; 2 Universidade Federal do ABC, Centro de Ciências Naturais e Humanas, São Bernardo do Campo, São Paulo, Brazil; National Center for Toxicological Research, UNITED STATES

## Abstract

There is a growing necessity to integrate morphological and genetic studies. This paper proposes a new technique that allows DNA extraction of arthropods while still keeping intact the entire morphology of the specimens. The technique uses Proteinase K to dissolve protein tissues and preserve the chitinous exoskeleton of specimens. The method is fast, cheap, non-toxic, and allows for good morphological preparations of specimens retaining much of their tridimensional structure. The methodology works fine with specimens preserved in different kinds of media, such as for dry (pinned) specimens, and specimens preserved in Ethanol. In addition, it allows the extraction of DNA from fresh specimens, as well as from specimens preserved for a long time. The technique works well for morphological studies alone, but allows the generation of an associated genomic library at an individual-scale. Among the advantages of the new technique is the possibility of extracting DNA from the entire specimen (necessary for the study of diseases transmitted by arthropod vectors), while still keeping the morphology intact for correct taxonomic identification. In addition, in comparison with methods that extract DNA from small tissue samples (e.g., from legs or wings), the method allows for the extraction of a larger amount of DNA and is better suited for small specimens.

## Introduction

Biological taxonomy has evolved considerably in recent times as different kinds of data are being integrated. Advances in techniques for DNA extraction and analysis of DNA sequence data caused an impact in taxonomy and systematics. Even though ways of dealing with the newly available data and integrating molecular and morphological data is often the subject of debate, the advances are unquestionable. For instance, the analysis of "DNA-barcoding" (technique that has strong supporters and critics) permitted the recognition of cryptic species not identified by morphological features [1, 2].

However, the speed and practicality of molecular techniques created a trend of studies without morphological support. In the begging of this century, the number of papers published in systematic journals dealing with molecular phylogenies alone exceeds the number of papers dealing with morphological and combined analyses. The proportion of combined studies (morphological and molecular data) is lower in insects than in other groups such as plants, fungi, bacteria and also other animals [3].

Current methods for DNA extraction from insect tissues are destructive, as the most common techniques macerate individuals (or parts of the individuals) to obtain DNA, and this imposes limits for its application. Taxonomists do not want to lose important parts of their rare specimens for molecular studies and molecular researchers need fresh materials with more quantity than the museum collections can normally offer. Also, extracting DNA from a small tissue sample (for instance, from one single leg) may not be able to provide a sufficient amount of DNA. This problem may be particularly important when dealing with small specimens. Therefore, the integration of morphology with molecular data would be more effectively accomplished if genomic data could be obtained of a specimen without destroying the morphology. Even if only one part is damaged, it can represent a great loss of information, especially for rare species or species that are already extinct. Molecular data obtained for a single or few specimens avoid the maceration of many individuals, but limit our ability to understand variability, and contrasting genetic versus morphological variability.

The most commonly used method for clarifying specimens in Entomology involves soaking the entire specimen or its part of interest in a 10% potassium hydroxide (KOH) solution. Then, KOH is removed by washing the specimens with acetic acid. The process can be slow and with unpredictable results. If the KOH solution is too concentrated, it may keep destroying the material due to the inefficacy of the acetic acid solution to completely neutralize the KOH. Also, techniques that use alkaline solutions such as KOH destroy nucleic acids. Often, the three-dimensional structure of the specimen is severely affected after transference from KOH to acetic acid and additional damage occur when the species are dehydrated in an ethanol series (80–98%), which is normally necessary before the specimen can be suitable for mounting in a microscope slide media such as Canada Balsam. The preservation of pinned specimens with volatile substances such as naphthalene also affects negatively the maintenance of the DNA, which becomes scarcer over time.

Our proposed technique for an integrative approach explores the fact that the chitinous exoskeleton of arthropods is resistant to proteinases, which will dissolve internal tissues (from which DNA can be extracted) and maintains the morphology of the exoskeleton. Previous analyses with beetles from museum collections raised expectations about extraction of DNA while keeping the morphology preserved in pinned specimens [4, 5]. For this study, we tested specimens stored under diverse conditions and for different amount of time. Also, we explored the clarification powers of the proteinase K solution as a method for preparation of the specimen for morphological studies in different media, such as microscope slides, ethanol, and glycerol. For many groups of insects, detailed morphological analysis is not possible for pinned specimens, and further preparation with clarification is necessary.

## Material and methods

### Specimens

Information on the specimens used in our study is described in the Table 1. This work did not involve use of human participants, specimens or tissue samples, or vertebrate animals, embryos or tissues. Also, no endangered or protected species were collected. Specific permissions, when required for specimen collections, were obtained by the responsible researcher of the institutions listed in Table 1. *Aedes aegypti* strain RED specimens, maintained in a biosafety level 2 (BSL-2) insectary facility in Institute of Biomedical Sciences from University of São Paulo, were gently donated by Margareth de Lara Capurro Guimarães. *Pullex irritans* and *Phlebotomus* sp. specimens were donated by Dr. Arlei Marcili being collected as part of an epidemiological investigation of infectious diseases transmitted by arthropods in the city of Bom Jesus dos Perdões, State of São Paulo, Brazil, and the protocol was approved by the Ethical Committee of Animal Use of the Faculty of Veterinary Medicine of the University of São Paulo. *Ae*. *aegypti* cultivated by Dr. Margareth de Lara Capurro Guimarães, and *Pullex irritans* and *Phlebotomus sp* specimens collected by Dr. Arlei Marcili, were obtained for purposes that are not related to this study and had approval and permissions needed in their own respective studies. *Geranomyia sp*. and *Chrysopilus balbii* were collected by the researchers described in Table 1 with permission of Instituto Chico Mendes de Conservação da Biodiversidade (ICMBio) and Instituto Brasileiro do Meio Ambiente e dos Recursos Naturais Renováveis (IBAMA). *Aphrophila chilena* specimen was borrowed from the National Museum of Natural History (Smithsonian Institution) with permission of Dr. Wayne Mathis (curator of the museum).

**Table 1:** Information on the studied specimens.

Taxon	Storage method	Collecting data	Age	Number of specimens
*Pulex irritans* (Siphonaptera)	Frozen material	Brazil, São Paulo, Bom Jesus dos Perdões (23.135 S 46.4656W), donated by Dr. Arlei Marcili in 2016.	Less than 1 year	7
*Phlebotomus s*p.(Diptera: Psychodidae)	Frozen material	Brazil, São Paulo, Bom Jesus dos Perdões (23.135 S 46.4656W), donated by Dr. Arlei Marcili in 2016.	Less than 1 year	11
*Aedes aegypti* (Diptera: Culicidae)	Frozen material	Strain RED specimens, donated by Dr. Margareth de Lara Capurro Guimarães in 2016.	Less than 1 year	5
*Geranomyia* sp. (Diptera: Limoniidae)	Ethanol 70°	Brazil, Santa Catarina, Joaçaba (27.1606S 51.5225W), Pinho LC and Muller GA, October 2012.	5 years	2
*Chrysopilus balbii (*Diptera: Rhagionidae*)*	Ethanol 70°	Brazil, São Paulo, Salesópolis (23.6500S 45.7333W), Papavero N, May 1968	14 years	1
*Aphrophila chilena* (Diptera: Limoniidae)	Pinned	Chile, Araucanía, Angol [37.1667S 72.1000W], Bullock DS, November 1929	87 years	1

### DNA extraction and PCR

The digestion buffer is composed of 200 mM Tris HCl, 250 mM NaCl, 25 mM EDTA, 0,5% de SDS and 400μg/mL of Proteinase K. All specimens included in this study were placed in a 1,5 mL sterile tube containing the amount of digestion buffer necessary to submerge the specimen (for this study was used on average 200–400 μL). The process usually happens at ambient temperature but the enzymatic digestion may be accelerated if incubated under temperature no higher than 56°C.

Afterwards, digestion buffer was collected with a micropipette and transferred to another sterile tube. The arthropod specimen was observed in a stereomicroscope to verify the clarification process. If necessary, new digestion buffer was added until obtaining appropriate clarification for morphological studies. Subsequently, digestion buffer was discarded and the specimen was covered with 80% of ethanol and kept at ambient temperature.

DNA was extracted from digestion buffer collected after 16 hours of specimen incubation with the employment of the DNasy extraction kit purchased from Qiagen, following manufacturer instructions, and was dissolved in a final volume of 30 μL. The protocol was deposited in protocols.io under link: dx.doi.org/10.17504/protocols.io.kkqcuvw. Concentration and quality of extracted DNA were determined by spectrophotometric evaluation of 1uL of sample, employing BioDrop μLITE^®^. An aliquot of 2 μL of each sample was analyzed by 1% agarose gel electrophoresis to verify DNA integrity. 100 ƞg (from samples with good quality genomic DNA) or 2 μL (degraded genomic DNA) of each specimen was submitted to PCR under conditions 94°C 2 min/35 cycles of 94°C 30 sec, 50°C 30 sec, 72°C 30 sec/72°C 5 min, with the GoTaq DNA polymerase from Promega, to amplify a 464 bp fragment corresponding to the 28S rRNA encoding gene from Diptera [6]. PCR products were analyzed by agarose gel electrophoresis stained with GelRed. The amplyfied products were purified with ThermoScientific PCR purification kit and submitted to Sanger's sequencing using Big Dye Terminator version 3.1 (ABI foster City, CA), according to manufacturer instructions. Sequences obtained were analyzed by Basic Local Alignment Search Tool (BLAST) [7].

## Results

### Morphological characterization

**Fig 1:**
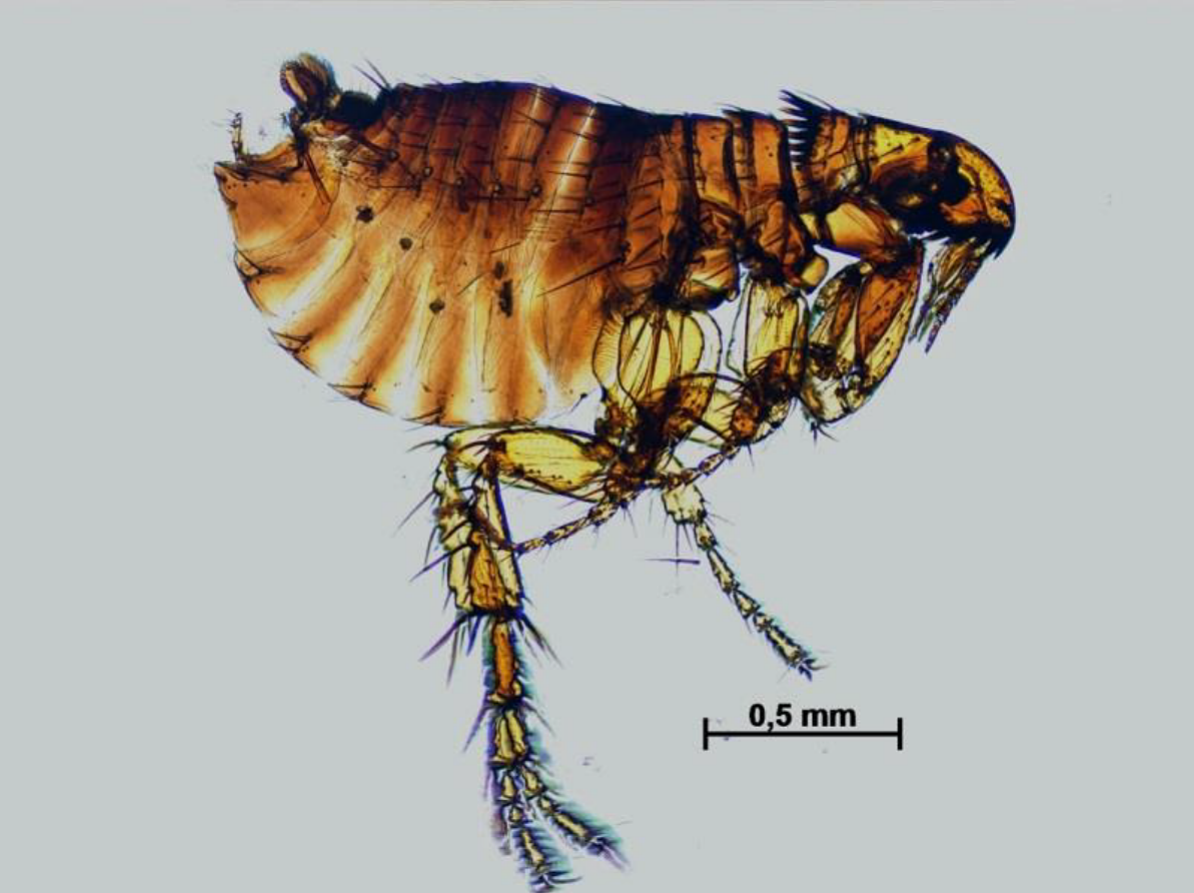
Clarified flea in lateral view.

The technique using proteinase k clarifies arthropods by the destruction of protein based structures. Differently from the method used in previous studies using pinned beetles vouchers from museum collections, which employed 250 mg/mL of proteinase K, 2% of SDS, 40 mM of DTT and 3 mM of CaCl_2_ [4,5], the amount of proteinase K decreased to 400 μg/mL and SDS to 0,5% in the DNA extraction buffer. This was mostly due to the smaller size of the insects included in our study. Also, DTT and CaCl_2_ were not included, reducing costs of the method and also avoiding the presence of reagents that could interfere with nucleic acid quality to be used in PCR reactions. Our proposed technique can clarify specimens with great amount of tissue. Robust insects such as fleas which normally require high concentrations of chemical reagent (KOH) can be easily clarified by the new technique with the same results and the same concentration of proteinase K (Fig 1).

**Fig 2:**
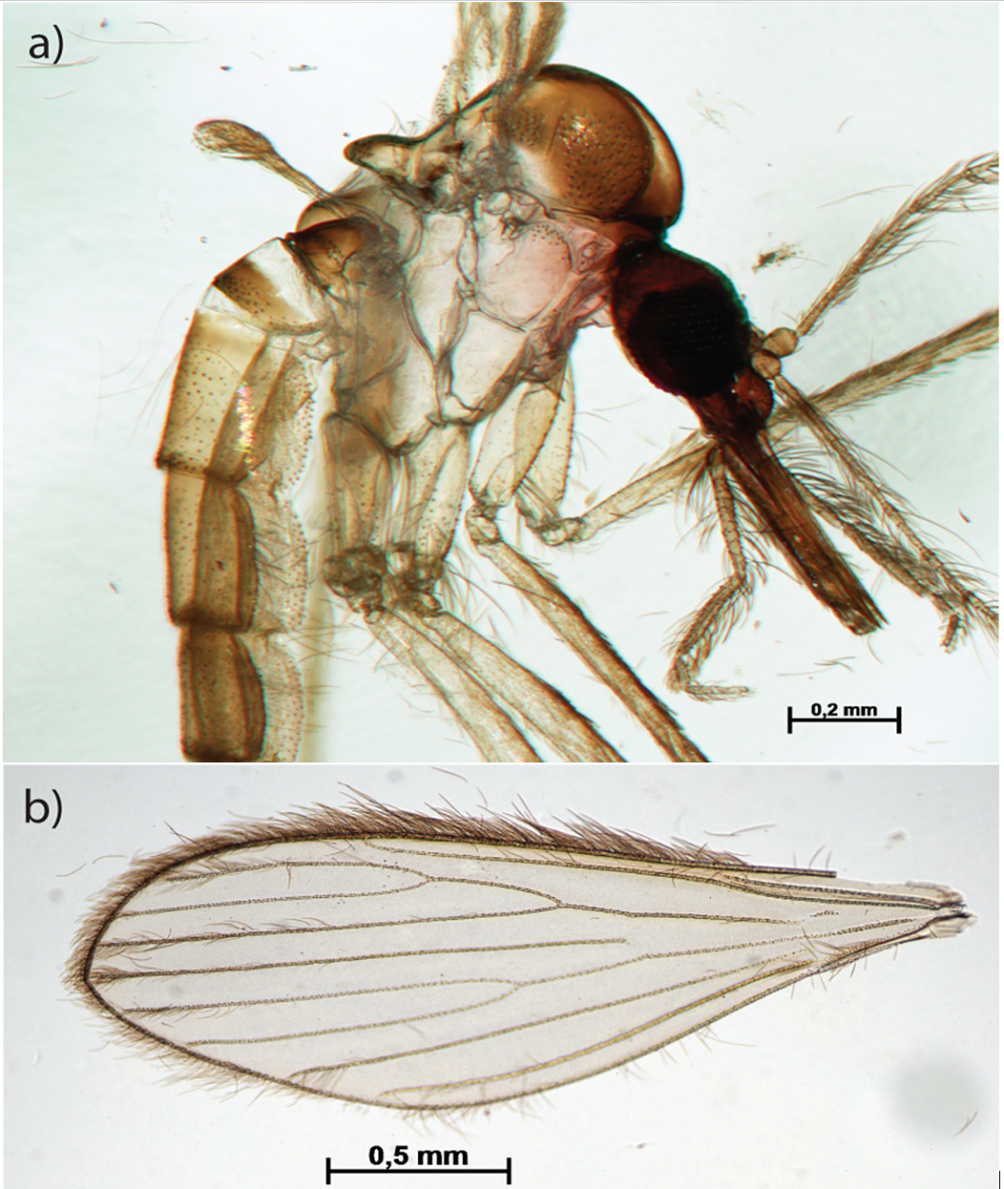
Clarified sand fly. Body in lateral view (a); wing (b).

The new technique also clarifies fragile specimens such as sand flies (Fig 2). Body parts such as antenna (Fig 2A) and setae (Fig 2B), which may have taxonomic importance, are well preserved. Although proteinase has a great effect in large protein based structures, it preserves even the most fragile chitin skeletal morphology.

**Fig 3:**
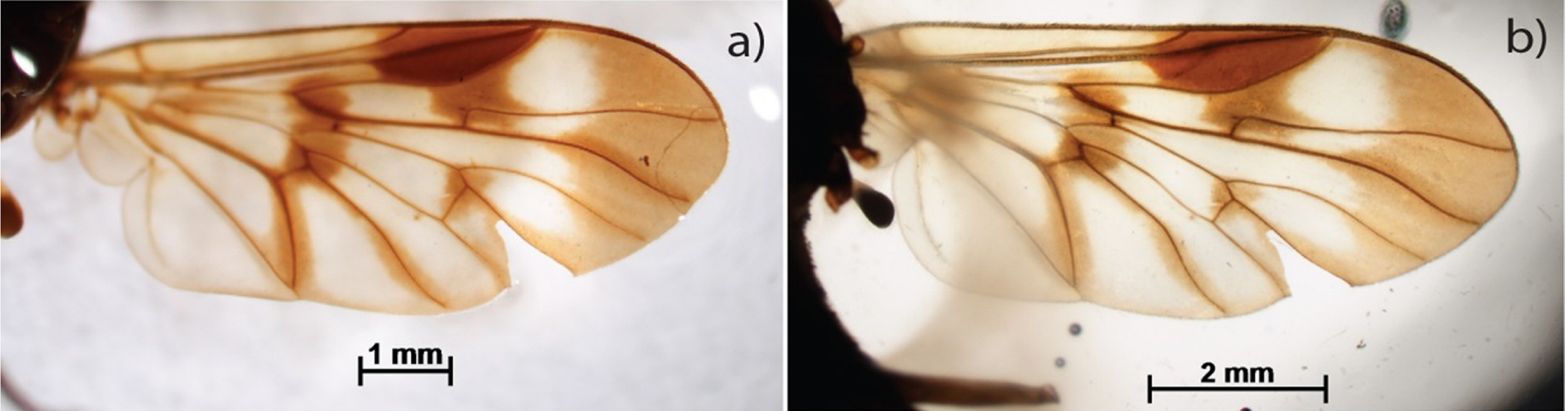
Chrysopilus balbii. Wing before (a) and after (b) clarification.

Color promoted by pigments constituted of protein changes after digestion buffer treatment. Pterostigma, which have taxonomic importance in some groups, is maintained (Fig 3A and 3B). It is always recommended to document the coloration of the specimens before the clarification because some colors may be altered or lost.

**Fig 4:**
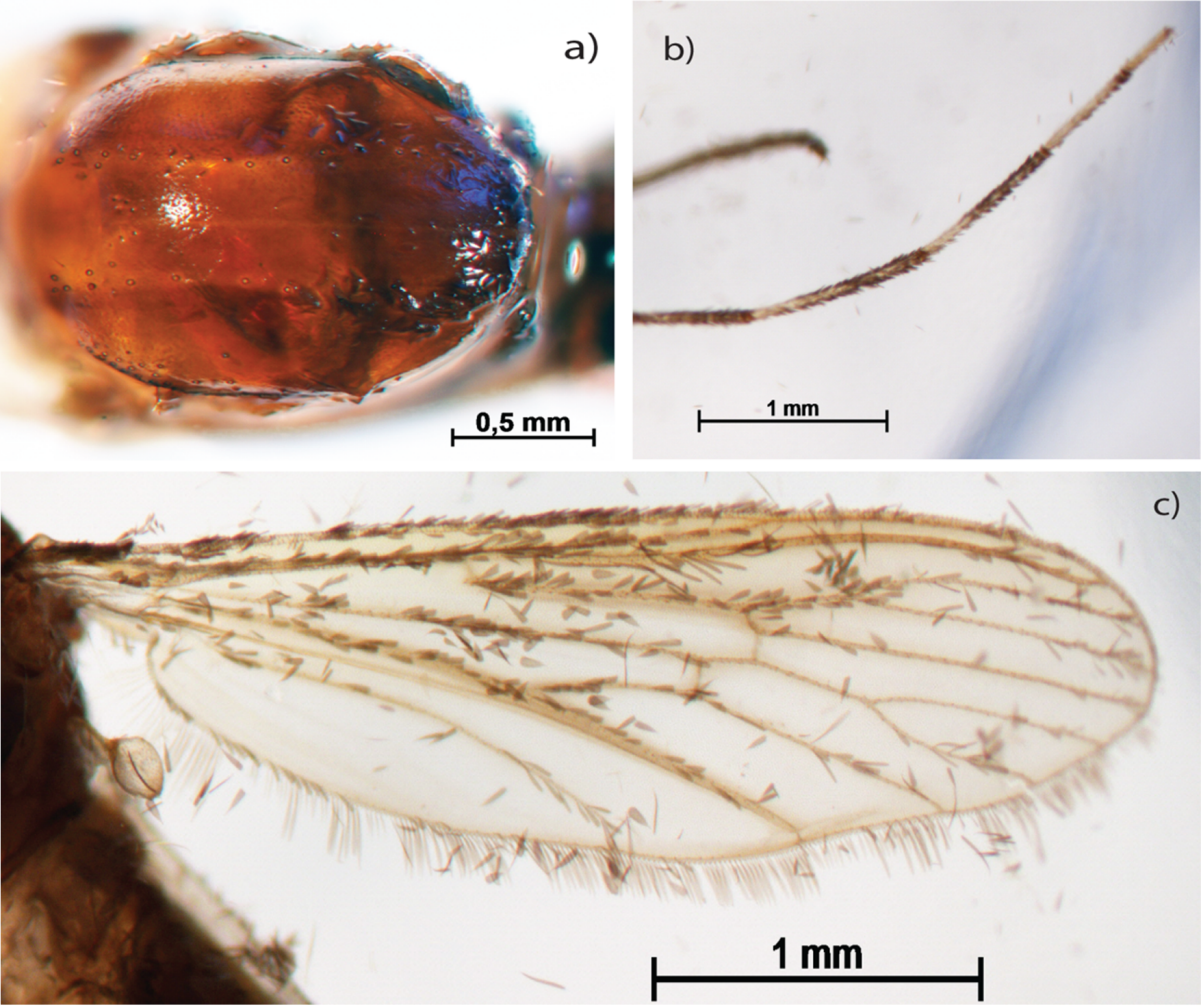
Clarified *Aedes aegypti*. Thorax in dorsal view (a); hind leg (b); wing in lateral view (c).

The method can distinguish the biochemical origin of pigments, as only pigments of protein based origin will be altered. The modifications of the color pattern of specimens of the genus *Aedes* illustrate this. The striped legs are characteristic of the genus and this pattern is preserved (Fig 4B) showing a non-protein based origin of these marks. But the characteristic markings at the dorsal surface of the thorax disappears (Fig 4A). Other features such as scales of wing are preserved (Fig 4C).

**Fig 5:**
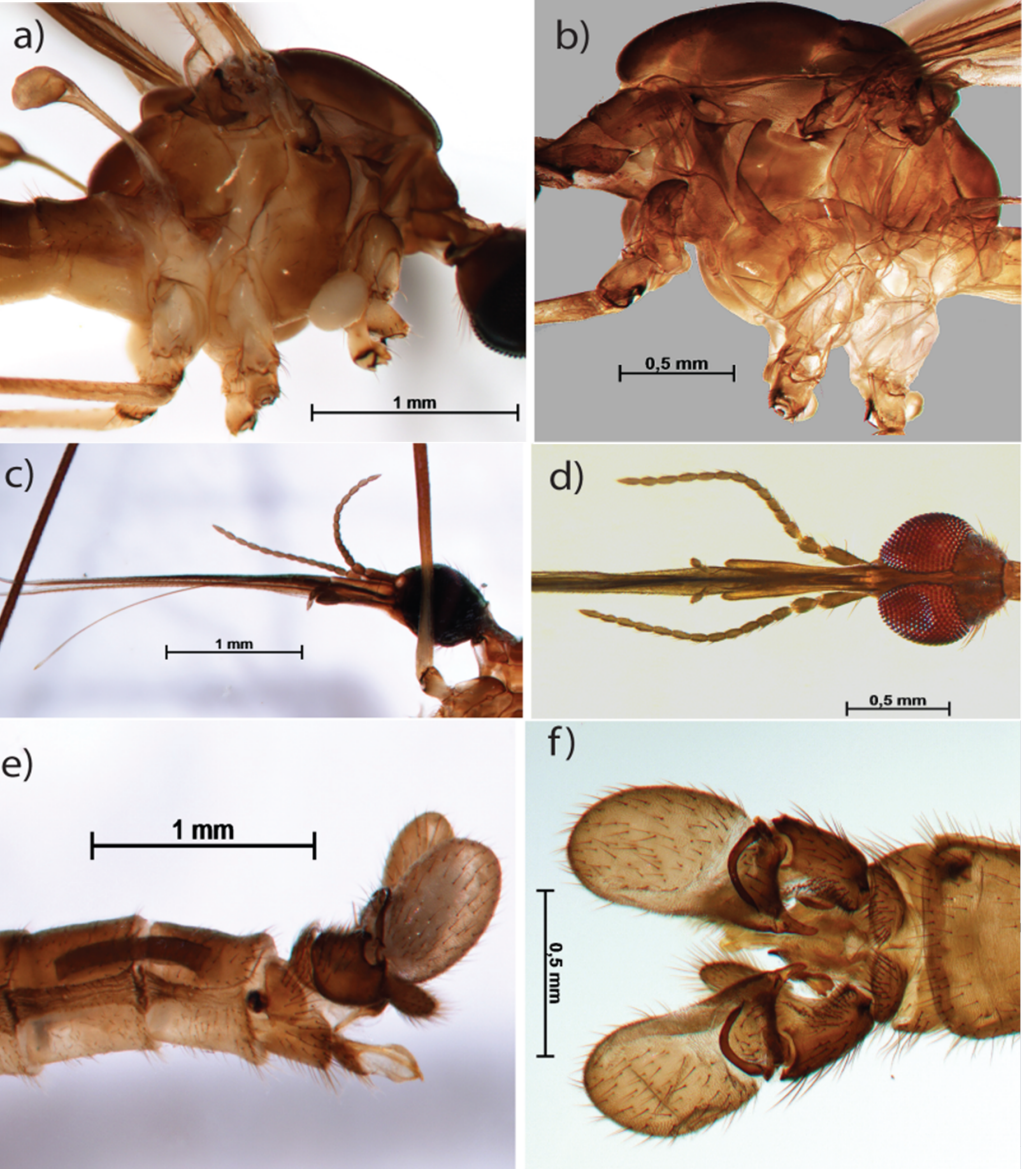
*Geranomyia* sp. Thorax in lateral view before (a) and after (b) clarification; head in lateral view before clarification (c) and in dorsal view after clarification (d); male abdomen in lateral view before clarification (e) and in dorsal view after clarification (f).

**Fig 6:**
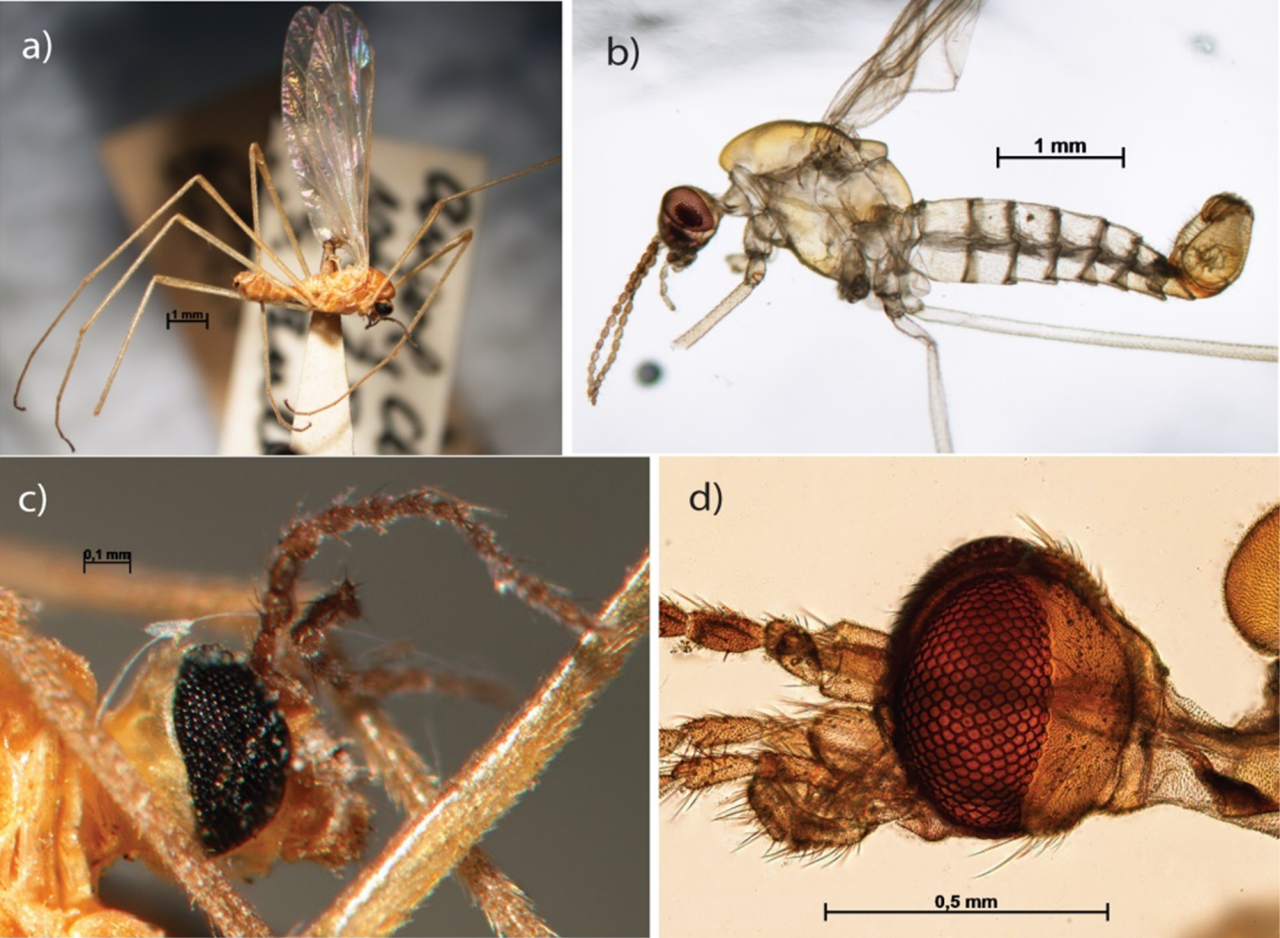
Aphrophila chilena. Habitus in lateral view before (a) and after (b) clarification; head in lateral view before (c) and after clarification (d).

Whether or not a research project includes the extraction of DNA in its objectives, it can take advantage from the proteinase K clarification technique. External morphology is maintained without much damage (Fig 5A and 5B), even for pinned specimens preserved for a long period of time (Fig 6A and 6B). The process also retains the tridimensional aspect of the structures (Figs 5C, 5D, 6C and 6D). Terminalia are well-preserved with good internal visualization (Figs 5E, 5F and 6B) that is very important for entomological studies.

### Genetic characterization

**Fig 7:**
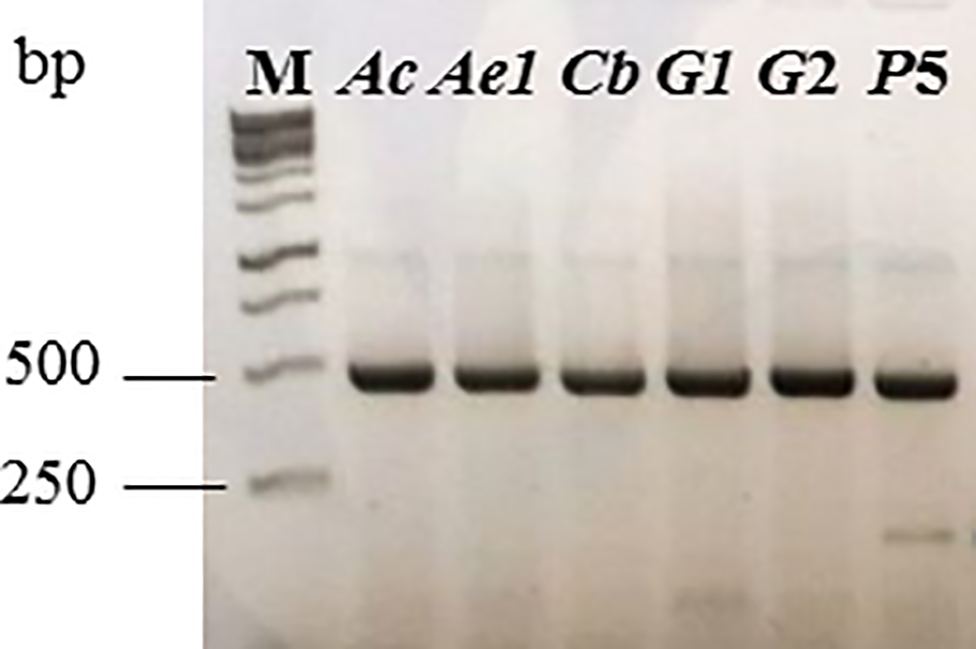
Analysis of PCR products stained with GelRed^®^ by 1% agarose gel electrophoresis. 10 μL of PCR reaction for amplification of the 464 bp fragment corresponding to the dipteran 28S rRNA encoding gene. M– 1 Kb ladder GeneO’ruler; *Ac–Aphrophila chilena*; *Ae*–*Aedes aegypti*; *Cb*–*Crysopilus balbii*; *G*–*Geranomyia* sp; *P*–*Phlebotomus* sp; numbers correspond to the specimens used in the PCR reaction according to Table 2.

Spectrophotometric analysis (Table 2) revealed good quality and quantity of the extracted DNA which was confirmed by PCR amplification of a 464 bp DNA fragment corresponding to 28S rRNA Diptera encoding gene (Fig 7). The DNA fragment was obtained for all Diptera specimens included in the study which were sequenced by Sanger method employing Big Dye Terminator version 3.1. The obtained sequences were analyzed by BLAST search and sequence specificity of well-studied species such as sand fly, and *Aedes* were confirmed. The new sequences obtained for two *Geranomyia* specimens (GenBank: MF996489; MF996490) presented high similarity to previous sequences for *Geranomyia canadensis* showing the efficiency of the technique. We also obtained the 464 bp of the 28S rRNA encoding gene for a specimen of the *Aphrophila chilena* (GenBank: MF996491). These are the first sequences of nuclear genes described to the genus *Aphrophila*.

**Table 2:** Spectrophotometric data of DNA extracted from the specimens included in the study.

Taxon	Storage method	Age	Specimen	DNA (ƞg/μL)	A260/ A280^a^	A260/ A230^a^
*Pulex irritans* (Siphonaptera)	Frozen material	< 1 year	1	176	2,03	1,97
			2	213	1,96	1,89
			3	184	1,79	1,92
			4	209	1,85	1,99
			5	176	1,81	1,90
			6	185	1,76	1,86
			7	193	1,78	1,95
*Phlebotomus sp* (Diptera: Psychodidae)	Frozen material	< 1 year	1	42,8	1,75	1,93
			2	44,5	1,79	2,03
			3	41,8	1,80	1,87
			4	41,1	1,74	1,99
			5	39,8	1,82	2,01
			6	50,9	1,77	1,85
			7	38,7	1,83	1,93
			8	43,5	1,80	1,89
			9	42,1	1,79	1,94
			10	41,4	1,81	2,08
			11	51,1	1,75	1,92
*Aedes aegypti* (Diptera: Culicidae)	Frozen material	< 1 year	1	63,6	1,78	1,97
			2	57,8	1,81	1,99
			3	42,1	1,76	2,10
			4	55,7	1,83	2,13
			5	51,2	1,73	1,87
*Geranomyia sp* (Diptera: Limoniidae)	Ethanol 70^0^	5 years	1	45,6	1,87	1,93
			2	37,8	1,78	1,99
*Chrysopilus balbii* (Diptera: Rhagionidae)	Ethanol 70^0^	14 years		87,1	1,75	1,87
*Aphrophila chilena* (Diptera: Limoniidae)	Pinned	87 years		172	1,81	1,96

^a^Absorbance ratios.

## Discussion

The main advantage of this new technique is the potential for integrative research which take into account genetic and morphological data. It is very significant to retain a preserved morphological voucher associated with genetic sequences. Barcoding initiatives, that are criticized by its limited approach [8, 9], can improve its explanatory power with morphological support by allowing access to a voucher specimen. The association of a single specimen with the molecular data obtained from it may have a great importance for medical studies. The new technique allows association of a specific individual with its DNA (and also with its RNA) contrasting with the usual methods that need many individual making a pool of organisms. For example, identification of new sylvatic vectors for protozoa parasites including *Plasmodium* sp and *Leishmania* sp is important for eco-epidemiological characterization of malaria and leishmaniasis. Also, studies of life-threatening arboviruses such as Dengue Fever, Chikungunya and Zika Virus, require a more accurate approach with individual precision, principally in identification of arthropods vectors in jungle cycles. The new technique allows the association of a unique individual with its precise localization and its specific strain. This aspect of the method cannot be overemphasized.

The technique works with old exemplars preserved under different methods. The clarified *Aphrophila chilena* specimen (Fig 5) was collected in 1923 and was preserved pinned in collections under the effect of substances such as naphthalene. Even so, we were able to extract DNA for PCR while keeping the entire specimens available for morphological studies. This opens the possibility for a new range of studies integrating morphology and genetics, even from groups for which only old specimens of museums are available. Even rare specimens that cannot be destroyed can be successfully preserved and analyzed genetically. The method is effective for specimens preserved in Ethanol, dry (pinned), or frozen.

In addition, the technique can reveal different metabolic (protein based or non-protein based) origins of color patterns which are difficult to distinguish based on morphological examination [10].

The technique also uses the non-toxic proteinase k contrasting with some toxic substances traditionally used in clarification of insects, such as cresol and KOH. By this enzymatic composition, the clarifier element is self-consuming and it allows a stable level of clarification. The other techniques such as KOH may not stop clarifying the specimen, leading to its complete destruction over time.

## References

[1] Pages N, Munoz-Munoz F, Talavera S, Sarto V, Lorca C, Nunez JI. Identification of cryptic species of Culicoides (Diptera: Ceratopogonidae) in the subgenus Culicoides and development of species-specific PCR assays based on barcode regions. Veterinary parasitology. 2009;165(3–4):298–310. Epub 2009/08/18. doi: 10.1016/j.vetpar.2009.07.020 . 19682796 10.1016/j.vetpar.2009.07.020

[2] Laurito M, Oliveira TM, Almiron WR, Sallum MA. COI barcode versus morphological identification of Culex (Culex) (Diptera: Culicidae) species: a case study using samples from Argentina and Brazil. Memorias do Instituto Oswaldo Cruz. 2013;108 Suppl 1:110–22. Epub 2014/01/30. doi: 10.1590/0074-0276130457 ; PubMed Central PMCID: PMC4109187. 24473810 10.1590/0074-0276130457 PMC4109187

[3] Bybee SM, Zaspel JM, Beucke KA, Scott CH, Smith BW, Branham MA. Are molecular data supplanting morphological data in modern phylogenetic studies? Systematic Entomology. 2010;35:4 doi: 10.1111/j.1365-3113.2009.00496.x

[4] Gilbert MT, Moore W, Melchior L, Worobey M. DNA extraction from dry museum beetles without conferring external morphological damage. PloS one. 2007;2(3):e272 Epub 2007/03/08. doi: 10.1371/journal.pone.0000272 ; PubMed Central PMCID: PMC1803022. 17342206 10.1371/journal.pone.0000272 PMC1803022

[5] Thomsen PF, Elias S, Gilbert MT, Haile J, Munch K, Kuzmina S, et al Non-destructive sampling of ancient insect DNA. PloS one. 2009;4(4):e5048 Epub 2009/04/02. doi: 10.1371/journal.pone.0005048 ; PubMed Central PMCID: PMC2660418. 19337382 10.1371/journal.pone.0005048 PMC2660418

[6] Tautz D, Hancock JM, Webb DA, Tautz C, Dover GA. Complete sequences of the rRNA genes of Drosophila melanogaster. Molecular biology and evolution. 1988;5(4):366–76. Epub 1988/07/01. doi: 10.1093/oxfordjournals.molbev.a040500 . 3136294 10.1093/oxfordjournals.molbev.a040500

[7] Altschul SF, Gish W, Miller W, Myers EW, Lipman DJ. Basic local alignment search tool. Journal of molecular biology. 1990;215(3):403–10. Epub 1990/10/05. doi: 10.1016/S0022-2836(05)80360-2 . 2231712 10.1016/S0022-2836(05)80360-2

[8] Trewick SA. DNA Barcoding is not enough: mismatch of taxonomy and genealogy in New Zealand grasshoppers (Orthoptera: Acrididae). Cladistics. 2008;24(2):15 doi: 10.1111/j.1096-0031.2007.00174.x

[9] Spooner DM. DNA barcoding will frequently fail in complicated groups: An example in wild potatoes. American journal of botany. 2009;96(6):1177–89. Epub 2009/06/01. doi: 10.3732/ajb.0800246 . 21628268 10.3732/ajb.0800246

[10] Shawkey MD, Morehouse NI, Vukusic P. A protean palette: colour materials and mixing in birds and butterflies. Journal of the Royal Society, Interface. 2009;6 Suppl 2:S221–31. Epub 2009/01/15. doi: 10.1098/rsif.2008.0459.focus ; PubMed Central PMCID: PMC2706479. 19141430 10.1098/rsif.2008.0459.focus PMC2706479

